# An Autocrine Negative Feedback Loop Inhibits Dictyostelium discoideum Proliferation through Pathways Including IP3/Ca^2+^

**DOI:** 10.1128/mBio.01347-21

**Published:** 2021-06-22

**Authors:** Yu Tang, Ramesh Rijal, David E. Zimmerhanzel, Jacquelyn R. McCullough, Louis A. Cadena, Richard H. Gomer

**Affiliations:** a Department of Biology, Texas A&M University, College Station, Texas, USA; Duke University

**Keywords:** *Dictyostelium*, cell density sensing, polyphosphate, cell proliferation, PLC/IP3/Ca^2+^, calcium signaling, inositol trisphosphate, quorum sensing

## Abstract

Little is known about how eukaryotic cells can sense their number or spatial density and stop proliferating when the local density reaches a set value. We previously found that Dictyostelium discoideum accumulates extracellular polyphosphate to inhibit its proliferation, and this requires the G protein-coupled receptor GrlD and the small GTPase RasC. Here, we show that cells lacking the G protein component Gβ, the Ras guanine nucleotide exchange factor GefA, phosphatase and tensin homolog (PTEN), phospholipase C (PLC), inositol 1,4,5-trisphosphate (IP3) receptor-like protein A (IplA), polyphosphate kinase 1 (Ppk1), or the TOR complex 2 component PiaA have significantly reduced sensitivity to polyphosphate-induced proliferation inhibition. Polyphosphate upregulates IP3, and this requires GrlD, GefA, PTEN, PLC, and PiaA. Polyphosphate also upregulates cytosolic Ca^2+^, and this requires GrlD, Gβ, GefA, RasC, PLC, IplA, Ppk1, and PiaA. Together, these data suggest that polyphosphate uses signal transduction pathways including IP3/Ca^2+^ to inhibit the proliferation of D. discoideum.

## INTRODUCTION

A longstanding idea in developmental biology is that the size of a tissue or group of cells, or the spatial density of a specific cell type, could be limited by an autocrine proliferation inhibitor, where the concentration of the inhibitor increases as the size of the tissue or cell group, or the density of cells, increases (1–8). The existence of autocrine proliferation inhibitors has been reported in mammalian tissues and organs, including skin (2), muscle (8), spleen (1), and liver (4), and the eukaryotic microorganism Dictyostelium discoideum (9). Although a considerable amount is known about signals and signal transduction pathways that promote cell proliferation, relatively little is known about autocrine proliferation-inhibiting signals and their signal transduction pathways.

Polyphosphate is a linear polymer of phosphate residues and is present in all kingdoms of life (10–12). In bacteria, polyphosphate functions in energy and phosphate storage (10) and potentiates both survival under some high-stress conditions (13) and biofilm formation (14, 15). In mammals, polyphosphate inhibits bone calcification (16) and the proliferation of leukemia cells (17), potentiates proinflammatory responses (18) and mTOR activation of plasma cells (19), accelerates blood coagulation (20), and induces apoptosis (21).

D. discoideum grows on soil surfaces and eventually overgrows its food supply and starves. D. discoideum accumulates extracellular polyphosphate as cells grow and proliferate (9). At cell densities corresponding to mid-log phase, the extracellular polyphosphate causes some cells to store rather than digest phagocytosed bacteria, possibly in anticipation of possible starvation (22). At very high cell densities, when the cells are about to starve, the accumulated extracellular polyphosphate reaches ∼150 μM. This concentration of polyphosphate contributes to the inhibition of cytokinesis (and, thus, cell proliferation) (9), possibly to prevent the formation of small cells. Therefore, just before starvation, the percentage of large cells with relatively large reserves of stored nutrients is increased (9).

Polyphosphate regulates the proliferation of D. discoideum by different signaling pathways depending on nutrient levels (23). In rich media, the loss of the G protein-coupled receptor GrlD, a metabotropic glutamate receptor-like receptor, partially reduced the sensitivity of cells to polyphosphate, and the loss of the small GTPase RasC did not reduce the sensitivity of cells to polyphosphate (23). However, under low-nutrient conditions, the loss of GrlD or RasC blocked the sensitivity of cells to polyphosphate (23).

The above-mentioned results suggest that polyphosphate uses a signal transduction pathway to inhibit D. discoideum proliferation under low-nutrient conditions. To elucidate additional signaling components in the polyphosphate proliferation inhibition pathway, we screened 52 available signal transduction pathway mutants for insensitivity to polyphosphate-induced proliferation inhibition under low-nutrient conditions. In combination with biochemical assays, we found evidence for a pathway involving inositol 1,4,5-trisphosphate (IP3) and cytosolic calcium that may mediate autocrine proliferation inhibition in *Dictyostelium.*

## RESULTS

### In addition to a G protein-coupled receptor and a Ras protein, a Ras GEF potentiates polyphosphate inhibition of cell proliferation.

**FIG 1:**
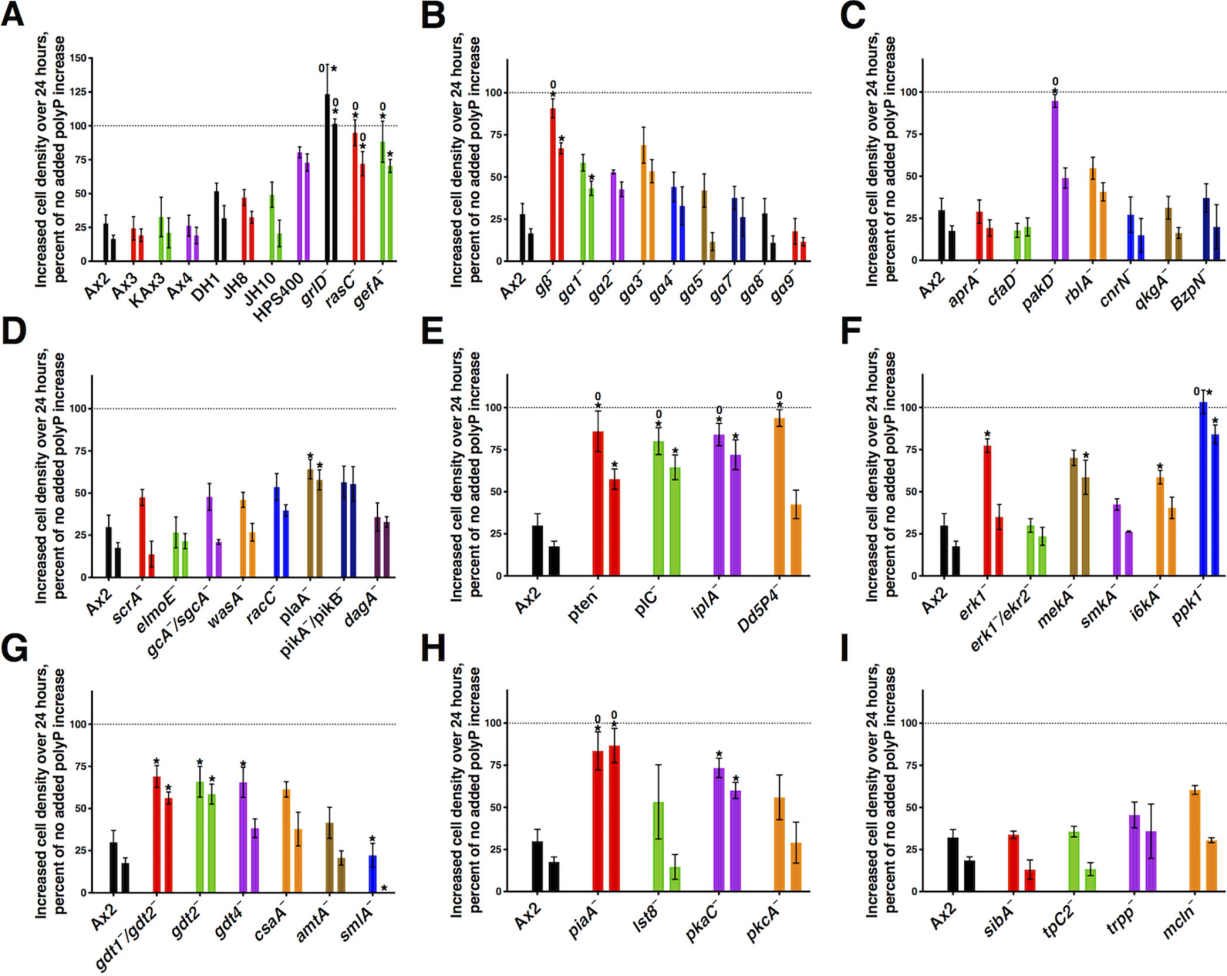
Some signal transduction pathway components are needed for polyphosphate (polyP) inhibition of proliferation in 25% HL5. The indicated cell lines were tested for proliferation with 0, 125, or 150 μM polyphosphate for 24 h. The increase in cell density over 24 h was normalized to the value with no added polyphosphate for the indicated strain. For each strain, the left bar is with 125 μM, and the right bar is with 150 μM polyphosphate. All values are means ± SEM (*n* ≥ 3 independent experiments). * indicates a *P* value of <0.05 compared to the parental wild-type cells with the same concentration of polyphosphate (by 2-way ANOVA, with multiple comparisons with Dunnett’s test within the panel). 0 indicates not significantly different from 100, and thus, the associated concentration of polyphosphate does not significantly inhibit proliferation in that mutant (by a two-tailed one-sample *t* test).

We previously observed that polyphosphate inhibits the proliferation of wild-type D. discoideum cells and that the loss of GrlD, RasC, or polyphosphate kinase 1 (Ppk1) reduces the ability of polyphosphate to inhibit proliferation (9, 23), suggesting the existence of a polyphosphate signal transduction pathway. To identify additional components of the polyphosphate proliferation inhibition pathway, 52 available mutants were screened for sensitivity to polyphosphate-induced proliferation inhibition under the low-nutrient condition of 25% HL5. The data were graphed in 9 groups: commonly used parental/wild-type cells (Ax2 to HPS400) and previously reported polyphosphate signal transduction pathway components (Fig. 1A), G protein subunits (Fig. 1B), AprA pathway components (Fig. 1C), selected cAMP pathway components (Fig. 1D), phospholipase C (PLC)/IP3 pathway components (Fig. 1E), mitogen-activated protein kinase (MAPK) pathway/polyphosphate synthesis pathway components (Fig. 1F), D. discoideum development-related proteins (Fig. 1G), TOR complex components/protein kinases (Fig. 1H), and mechanotransduction components (Fig. 1I). The initial cell density was 1.5 × 10^6^ cells/ml, and cells were counted 24 h later. The data were plotted as 100 × (density with polyphosphate − 1.5 × 10^6^ cells/ml)/(density with no added polyphosphate − 1.5 × 10^6^ cells/ml). This value would then be 100 if the polyphosphate had no effect on cell proliferation and 0 if the polyphosphate completely inhibited cell proliferation. Compared to no added polyphosphate, 125 μM and 150 μM polyphosphate reduced the increase in the cell density of Ax2 wild-type cells to ∼30% and ∼18%, respectively (Fig. 1A). At 24 h, the density of the Ax2 cells with no polyphosphate was 3.9 × 10^6^ ± 0.1 × 10^6^ cells/ml (mean ± standard error of the mean [SEM]) (*n* = 7) (see Table S2 in the supplemental material), so the 18% cell density increase at 24 h represents a change in the doubling time from the control value of 17.7 ± 0.7 h to 81.3 ± 16.7 h. Polyphosphate also reduced the proliferation of all the other commonly used parental/wild-type strains (Fig. 1A). The proliferation of these strains in the absence of added polyphosphate, and all of the mutant strains described below, is shown in Table S2.

10.1128/mBio.01347-21.6 TABLE S2 Some mutants have abnormal proliferation in 25% HL5. The indicated cell lines were tested for proliferation for 24 h. Cells were cultured in 25% HL5, starting with 1.5 × 10^6^ cells/ml. All values are means ± SEM (*n* ≥ 3 independent experiments). *, *P* < 0.05; **, *P* < 0.01; ***, *P* < 0.001 (by a two-tailed *t* test compared to the parental cell line). Download Table S2, DOCX file, 0.02 MB. Copyright © 2021 Tang et al. 2021 Tang et al. https://creativecommons.org/licenses/by/4.0/ This content is distributed under the terms of the Creative Commons Attribution 4.0 International license.

As previously reported, compared to Ax2 cells, cells lacking the putative polyphosphate receptor GrlD (23) or the Ras protein RasC (24) showed abolished sensitivity (no significant difference compared to no added polyphosphate by a *t* test) to 125 and 150 μM polyphosphate (Fig. 1A). Cells lacking GefA, a Ras guanine nucleotide exchange factor (GEF) for RasC but not RasB, RasD, or Rap1 (25), also showed reduced sensitivity to polyphosphate (Fig. 1A). The density of cells lacking RasG after 24 h was 80% ± 18% (mean ± SEM) (*n* = 3) of the initial cell density, suggesting that cells lacking *rasG* (*rasG*^−^ cells) do not grow in 25% HL5.

### The Gβ subunit potentiates polyphosphate inhibition of cell proliferation.

Cells lacking the heterotrimeric G protein subunit Gβ (26) showed reduced sensitivity to polyphosphate inhibition of cell proliferation (Fig. 1B). Cells lacking Gα2, -3, -4, -5, -7, -8, or -9 did not have significantly abnormal sensitivity to polyphosphate. Cells lacking Gα1 (27) showed increased sensitivity to polyphosphate at 150 μM compared to their parental strain HPS400. Whereas cells lacking the putative receptor GrlD appeared to be completely insensitive to polyphosphate, none of the Gα mutants showed complete insensitivity. Comparing the values for *grlD*^−^ cells in Fig. 1A to those for the G protein mutants in Fig. 1B, although at 125 μM, the difference for *g*β^−^ was not significant, at 150 μM, the differences for *g*β^−^ were significant, with a *P* value of <0.01 (by *t* tests). These results suggest that there is an additional pathway downstream of GrlD that does not involve the single characterized Gβ in *Dictyostelium* (26) and that GrlD may activate multiple Gα subunits or untested Gα subunits.

### The AprA pathway component PakD potentiates polyphosphate inhibition of cell proliferation.

AprA is a secreted autocrine proliferation repressor and chemorepellent (28). Compared to their parental Ax2 cells, cells lacking the AprA pathway component PakD (a p21-activated kinase family member) (29) showed reduced sensitivity to polyphosphate (Fig. 1C). Compared to wild-type cells, cells lacking AprA, CfaD (a secreted factor that binds to AprA and then slows cell proliferation) (30), RblA (a retinoblastoma ortholog) (31), CnrN (a phosphatase and tensin homolog [PTEN]-like phosphatase involved in AprA sensing) (32–34), QkgA (a leucine-rich repeat [LRR] kinase family protein that is required for AprA-induced proliferation inhibition and chemorepulsion) (35), or BzpN (a transcription factor that is required for AprA-induced proliferation repression) (36) did not show significantly abnormal sensitivities to polyphosphate (Fig. 1C), indicating that many AprA pathway components are not used by polyphosphate to inhibit proliferation.

### The cAMP response component PlaA potentiates polyphosphate inhibition of cell proliferation.

Gβ and Gα2 mediate cAMP signaling in developing cells (37, 38). Compared to their parental Ax3 cells, cells lacking the cAMP chemoattraction pathway component phospholipase A2 PlaA (39) showed reduced sensitivity to 125 μM or 150 μM polyphosphate (Fig. 1D). Cells lacking ScrA (an adaptor protein that regulates actin polymerization) (40), ElmoE (an engulfment and cell motility protein, which transduces signals from chemoattractant receptors to the cytoskeleton) (41), GcA and SgcA (membrane-bound and soluble guanylyl cyclases, respectively) (42), WasA (an adaptor protein that regulates actin polymerization) (43), PikA and -B phosphatidylinositol kinases (44), or DagA (the cytosolic regulator of adenylate cyclase) (45) did not show significantly abnormal sensitivity to polyphosphate (Fig. 1D), indicating that many of the components that mediate cAMP chemoattraction are dispensable for polyphosphate to inhibit proliferation.

### The PLC/IP3 pathway components PTEN, PLC, IplA, and Dd5P4 potentiate polyphosphate inhibition of cell proliferation.

Cells lacking PTEN (46), PLC (47), the inositol 1,4,5-trisphosphate (IP3) receptor-like protein IplA (48), or the inositol 5-phosphatase 4 Dd5P4 (49) showed abolished sensitivity to 125 μM polyphosphate, and compared to their parental Ax2 or DH1 cells, cells lacking PTEN, PLC, or IplA showed reduced sensitivity to 150 μM polyphosphate (Fig. 1E). PTEN catalyzes the conversion of phosphatidylinositol (3-5)-trisphosphate (PIP3) to phosphatidylinositol (4,5)-bisphosphate (PIP2) (50), and PLC catalyzes the hydrolysis of PIP2 to diacylglycerol (DAG) and IP3 (50). IplA is a potential IP3 receptor in D. discoideum (48). Dd5P4 dephosphorylates PIP3, PIP2, and IP3 (51). These results suggest that the PLC/IP3 pathway affects or is involved in polyphosphate inhibition of cell proliferation and that IP3 might be a second messenger in the polyphosphate signal transduction pathway.

### The MAPK/Erk pathway components Erk1 and MekA potentiate polyphosphate inhibition of cell proliferation.

Compared to their parental KAx3 or JH10 cells, cells lacking the extracellular signal-regulated kinase Erk1 (52) or the Erk1 kinase MekA (53) showed reduced sensitivity to 125 μM or 150 μM polyphosphate inhibition of cell proliferation (Fig. 1F). Deleting the suppressor of MekA, SmkA (53), did not significantly alter sensitivity to polyphosphate (Fig. 1F). These results suggest that the MekA-Erk1 pathway is involved in polyphosphate proliferation inhibition.

### The polyphosphate synthesis pathway components I6kA and Ppk1 potentiate polyphosphate inhibition of cell proliferation.

The inositol phosphate kinase I6kA does not appear to affect intracellular polyphosphate levels at cell densities below ∼1 × 10^7^ cells/ml but plays a role in upregulating intracellular polyphosphate at cell densities of ≥2 × 10^7^ cells/ml (9). The polyphosphate kinase Ppk1 is essential for intracellular polyphosphate production at all cell stages (12). Compared to their parental Ax2 cells, cells lacking I6kA showed reduced sensitivity to 125 μM polyphosphate. Cells lacking Ppk1 showed abolished sensitivity to 125 μM polyphosphate and strongly reduced sensitivity to 150 μM polyphosphate (Fig. 1F). The correlation between intracellular polyphosphate synthesis and sensitivity to extracellular polyphosphate suggests that intracellular polyphosphate plays a role in polyphosphate inhibition of cell proliferation.

### The development-related Gdt proteins potentiate polyphosphate inhibition of cell proliferation.

Members of the growth-differentiation transition family of proteins (Gdts) are *Dictyostelium*-specific tyrosine kinase-like proteins, classified by their sequence similarity and their participation in development (54). Gdt1 and Gdt2 are negative regulators of the *Dictyostelium* growth-differentiation transition process (54, 55), but there is no report about the function of Gdt4 yet. Compared to their parental Ax4 cells, cells lacking growth-differentiation transition family member 2, or both Gdt1 and -2, showed reduced sensitivity to both 125 μM and 150 μM polyphosphate (Fig. 1G). Cells lacking Gdt4 had reduced sensitivity to 125 μM polyphosphate. Cells lacking the protein contact site A CsaA (56) or the ammonium transporter AmtA (57) did not show significantly altered sensitivity to polyphosphate. These results suggest that Gdt2 and Gdt4 may play a role in cell proliferation.

### The cell aggregate size regulator SmlA attenuates polyphosphate inhibition of cell proliferation.

The small-aggregate formation protein SmlA regulates the size of cell aggregates and fruiting bodies during development by inhibiting the extracellular accumulation of the group size-regulating factor counting factor (58, 59). Compared to their parental strain DH1, for unknown reasons, cells lacking SmlA showed increased sensitivity to 125 μM polyphosphate and appeared to be hypersensitive to 150 μM polyphosphate (after 24 h, this polyphosphate concentration caused the cell density to decrease from 1.5 × 10^6^ cells/ml to 1.2 × 10^6^ ± 0.2 × 10^6^ cells/ml [mean ± SEM] [*n* = 4]) (Fig. 1G).

### The TORC2 component PiaA and the protein kinase PKA-C potentiate polyphosphate inhibition of cell proliferation.

*Dictyostelium* Tor complex 2 (TORC2), composed of Tor, PiaA, Lst8, and Rip3, regulates adenylyl cyclase ACA (60, 61) and protein kinase B/Akt activation (60, 62) and is essential for cell aggregation (60, 63). Cells lacking the TORC2 component PiaA (Rictor) but not Lst8 showed abolished sensitivity to both 125 μM and 150 μM polyphosphate, suggesting that PiaA is an essential component of the polyphosphate proliferation inhibition pathway (Fig. 1H). Compared to their parental JH10 cells, cells lacking the cAMP-dependent protein kinase catalytic subunit PKA-C (64) showed reduced sensitivity to polyphosphate inhibition of cell proliferation, suggesting that cAMP might be a messenger in the polyphosphate proliferation inhibition pathway (Fig. 1H). Compared to wild-type cells, cells lacking Lst8 or protein kinase C (PKCA) did not show significantly abnormal sensitivities to polyphosphate (Fig. 1H), indicating that some components of the PKCA pathway are dispensable for polyphosphate to inhibit proliferation.

### Four mechanotransduction components do not significantly affect polyphosphate inhibition of cell proliferation.

Testing a variety of other signal transduction pathway components, we observed that cells lacking the mechanotransduction components SibA (an integrin beta-like protein) (65), TPC2 (two-pore calcium channel protein 2) (65), TrpP (the transient receptor potential cation channel protein) (65), or Mcln (an ortholog of mucolipin) (65) did not show significantly altered sensitivities to polyphosphate compared to their parental DH1 cells (Fig. 1I). These results suggest that many components of the mechanotransduction pathway are dispensable for polyphosphate to inhibit proliferation.

### Gβ, GefA, PTEN, PLC, IplA, Ppk1, and PiaA potentiate polyphosphate inhibition of cell proliferation in both 25% and 100% HL5.

With two-way analysis of variance (ANOVA) (multiple comparisons with Dunnett’s test), cells lacking GrlD, Gβ, GefA, RasC, PTEN, PLC, IplA, Ppk1, or PiaA showed strongly reduced sensitivity to both 125 and 150 μM polyphosphate (indicated by * in Fig. 1A, B, and E to H) and showed abolished sensitivity (no statistical difference [by a one-sample *t* test] with 100% proliferation) to 125 μM polyphosphate (indicated by 0) (Fig. 1A, B, E, F, and H). They were thus chosen for further tests.

To further test the effects of the genes encoding Gβ, GefA, PTEN, PLC, IplA, Ppk1, and PiaA on the cells’ sensitivity to polyphosphate, mutant and available complemented strains were tested for sensitivity to polyphosphate with a more extensive dose-response curve in 25% HL5 (Fig. S1) (these assays were previously done for GrlD and RasC [17]). Compared to their respective parental wild-type cells, cells lacking Gβ, GefA, PTEN, PLC, IplA, Ppk1, or PiaA showed reduced sensitivity to physiological levels of polyphosphate (150 μM or lower) (Fig. S1). The 50% inhibitory concentrations (IC_50_s) of polyphosphate proliferation inhibition of these knockout mutant strains were higher than that of parental wild-type cells in 25% HL5 (Table 1). Expressing PTEN in *pten*^−^ cells and PLC in *plC*^−^ cells rescued or partially rescued the decreased sensitivity to polyphosphate (Table 1 and Fig. S1C and D).

**TABLE 1:** Deletion of some potential polyphosphate pathway components increases the IC_50_ for polyphosphate inhibition of proliferation^*a*^

Strain	Mean IC_50_ (μM) ± SEM in 25% HL5	Mean IC_50_ (μM) ± SEM in 100% HL5
Ax2	106 ± 3	117 ± 10
DH1	121 ± 16	102 ± 9
*g*β^−^	173 ± 13	>200
*gefA* ^−^	160 ± 5	177 ± 35
*pten* ^−^	178 ± 15**	>200
*pten* ^−^ */pten-GFP*	127 ± 8@	91 ± 7
*plC* ^−^	168 ± 6**	>200
*plC* ^−^ */plC*	124 ± 3@	130 ± 3
*iplA* ^−^	192 ± 8***	188 ± 41
*ppk1* ^−^	168 ± 5***	189 ± 48
*piaA* ^−^	167 ± 10***	>200

a IC_50_s were calculated from the data in Fig. S1 and S2 in the supplemental material, using Prism with nonlinear regression (sigmoidal dose-response, variable slope, and the top constrained to 100). All values are means ± SEM (*n* ≥ 3 independent experiments). *, *P* < 0.05; **, *P* < 0.01; ***, *P* < 0.001 (compared to the parental wild-type strain Ax2 or DH1 [by a two-tailed *t* test or one-way ANOVA followed by Tukey’s test among DH1, *plC*^−^, and *plC*^−^*/plC* cells or among Ax2, *pten*^−^, and *pten*^−^*/pten-GFP* cells]). @, *P* < 0.001 (compared to *plC*^−^ or *pten*^−^ cells [by one-way ANOVA with Tukey’s test among DH1, *plC¯*, and *plC*^−^*/plC* cells or among Ax2, *pten*^−^, and *pten*^−^*/pten-GFP* cells]).

10.1128/mBio.01347-21.1 FIG S1 Some mutants have abnormal sensitivities to polyphosphate in 25% HL5. The indicated cell lines were tested for proliferation in 25% HL5 with the indicated concentrations of polyphosphate for 24 h. The increase in the cell density over 24 h was normalized to the increase with no added polyphosphate. Curve fits were done using Prism with nonlinear regression (sigmoidal dose-response, variable slope, and the top constrained to 100). Values are means ± SEM (*n* ≥ 3). Download FIG S1, TIF file, 1.4 MB. Copyright © 2021 Tang et al. 2021 Tang et al. https://creativecommons.org/licenses/by/4.0/ This content is distributed under the terms of the Creative Commons Attribution 4.0 International license.

10.1128/mBio.01347-21.2 FIG S2 Some mutants have abnormal sensitivities to polyphosphate in 100% HL5. The indicated cell lines were tested for proliferation in 100% HL5 as described in the legend of Fig. S1 in the supplemental material. Values are means ± SEM (*n* ≥ 3). Download FIG S2, TIF file, 1.4 MB. Copyright © 2021 Tang et al. 2021 Tang et al. https://creativecommons.org/licenses/by/4.0/ This content is distributed under the terms of the Creative Commons Attribution 4.0 International license.

To determine if these proteins are also involved in the polyphosphate signal transduction pathway under nutrient-rich conditions, the corresponding knockout strains were tested for sensitivity to polyphosphate with dose-response curves in 100% HL5 (Fig. S2). In 100% HL5, compared to parental wild-type cells, cells lacking Gβ, GefA, PTEN, PLC, IplA, Ppk1, or PiaA also showed reduced sensitivity to polyphosphate (Fig. S2). In 100% HL5, the proliferation inhibition curve fits for *g*β^−^ and *pten*^−^ cells could not be generated, and the curve fits for *gefA*^−^, *plC*^−^, *iplA*^−^, *ppk1*^−^, and *piaA*^−^ cells were ambiguous. The IC_50_s of polyphosphate proliferation inhibition of these knockout mutant strains were higher than that of parental wild-type cells (Table 1). Expressing PTEN in *pten*^−^ cells and PLC in *plC*^−^ cells appeared to partially rescue or rescue the decreased sensitivity to polyphosphate (Table 1 and Fig. S2C and D). Together, these results support the idea that Gβ, GefA, PTEN, PLC, IplA, Ppk1, and PiaA affect the polyphosphate proliferation inhibition signal transduction pathway under both low- and high-nutrient conditions.

### Gβ, PTEN, PLC, IplA, Ppk1, and PiaA affect cell proliferation.

**FIG 2:**
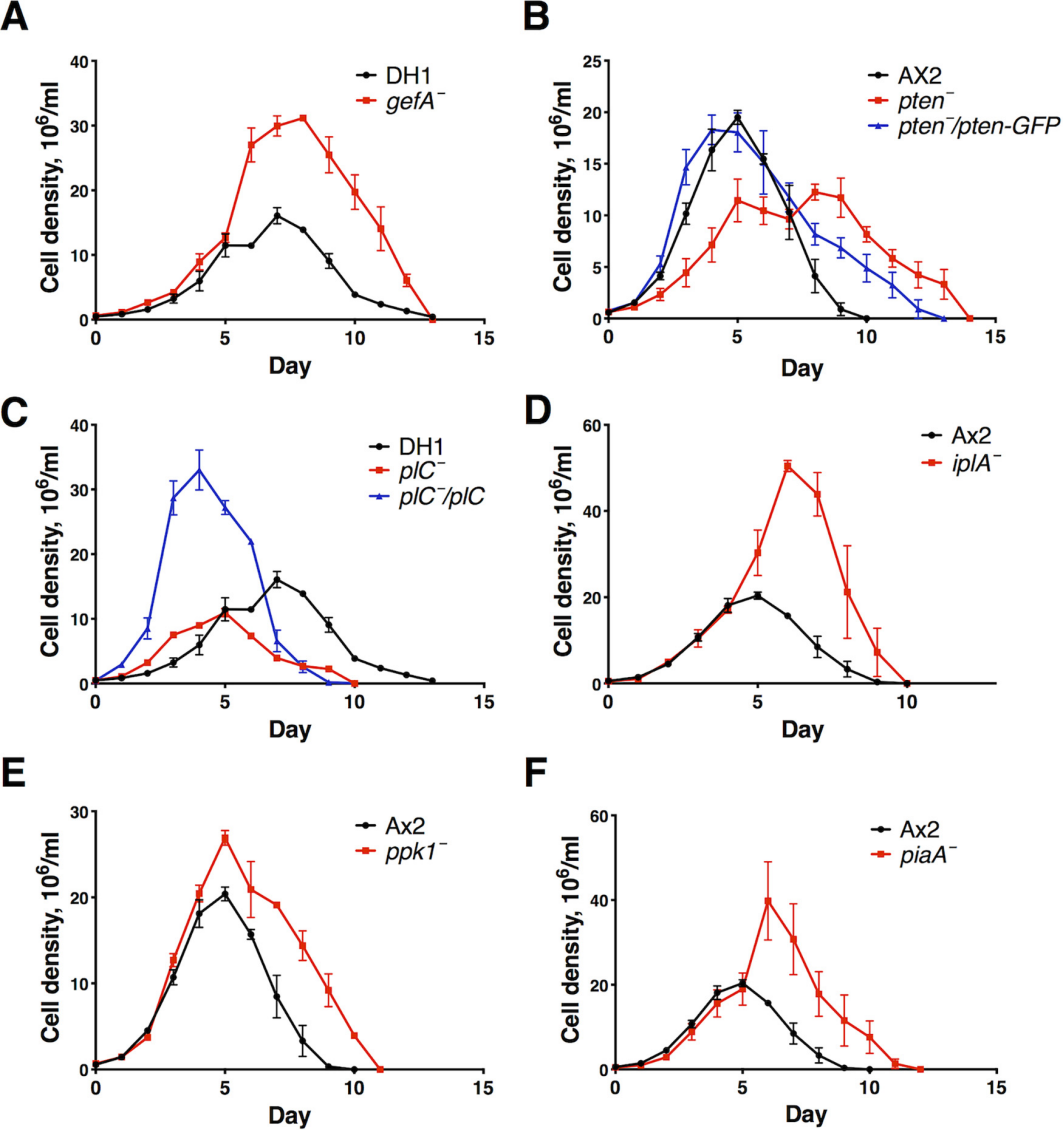
Some mutants have abnormal growth curves in HL5. Log-phase cells were grown in a liquid shaking culture starting at ∼5 × 10^5^ cells/ml and counted daily. All values are means ± SEM (*n* ≥ 3 independent experiments). Data and statistical analysis are shown in Table 2.

To assess the effect of the disruption of these genes on general cell proliferation, we assayed proliferation curves of the above-described strains in 100% HL5 in a shaking culture (Fig. 2), except for *g*β^−^ cells, which were assayed previously (66). The doubling times at a low cell density (∼0.5 × 10^6^ to 6 × 10^6^ cells/ml) and a high cell density (6 × 10^6^ cells/ml to the maximal cell density or plateau) were calculated. At low cell densities, where the extracellular polyphosphate concentration is expected to be low, cells lacking PTEN or Ppk1 had a longer doubling time than Ax2 cells (Table 2), and cells lacking Gβ or PLC had a shorter doubling time than the parental wild-type DH1 cells (66). Expressing PTEN in *pten*^−^ cells rescued the long-doubling-time phenotype, and expressing PLC in *plC*^−^ cells further shortened the doubling time (Table 2), possibly because too little or too much PLC potentiates cell proliferation. At high cell densities, where the extracellular polyphosphate concentration is expected to be high, cells lacking IplA, Ppk1, or PiaA had shorter doubling times than Ax2 cells (66), and cells lacking PLC had a longer doubling time than DH1 cells (Table 2). Expressing PLC in *plC*^−^ cells caused a shorter doubling time than in DH1 cells (Table 2). These data suggest that PTEN and Ppk1 promote cell proliferation at low cell densities; PLC promotes cell proliferation, and IplA, Ppk1, and PiaA slow cell proliferation at high cell densities. The maximal cell density is abnormally high in cells lacking Gβ, GefA, IplA, Ppk1, or PiaA (66) (Fig. 2A, D, E, and F and Table 2) and is abnormally low in cells lacking PTEN or PLC (Fig. 2B and C and Table 2). Expressing PTEN in *pten*^−^ cells and PLC in *plC*^−^ cells rescued or reversed the phenotype (Fig. 2B and C and Table 2). These data suggest that these genes affect the proliferation of D. discoideum cells.

**TABLE 2:** Deletion of some potential polyphosphate pathway components alters the doubling time and maximal cell density^*a*^

Strain	Mean doubling time (h) ± SEM		Maximal density (10^6^ cells/ml)
	Low density	High density	
Ax2	16.3 ± 1.1	32.4 ± 1.7	21.8 ± 0.7
DH1	29.3 ± 2.1	26.0 ± 2.7	16.1 ± 1.2
*gefA* ^−^	26.3 ± 1.2	27.2 ± 1.2	31.8 ± 0.5**
*pten* ^−^	29.4 ± 4.4*	33.8 ± 4.0	13.8 ± 1.1***
*pten* ^−^ */pten-GFP*	17.6 ± 1.3@	32.9 ± 3.5	22.5 ± 0.7@
*plC* ^−^	21.2 ± 0.9*,@	41.8 ± 5.0@	11.0 ± 0.6*
*plC* ^−^ */plC*	9.0 ± 0.6**,@	20.9 ± 1.2	33.0 ± 3.1*,@
*iplA* ^−^	14.9 ± 0.5	25.2 ± 1.7*	52.0 ± 1.3***
*ppk1* ^−^	19.9 ± 0.9*	25.3 ± 1.1*	27.3 ± 0.7**
*piaA* ^−^	19.4 ± 1.6	23.7 ± 2.2*	38.4 ± 9.1

a For the data in Fig. 2, doubling times were calculated for low cell densities (0.5 × 10^6^ to 6 × 10^6^ cells/ml) and high cell densities (6 × 10^6^ cells/ml to the maximal density reached). Values are means ± SEM (*n* ≥ 3 independent experiments). *, *P* < 0.05; **, *P* < 0.01; ***, *P* < 0.001 (compared to their parental strains [by a *t* test or one-way ANOVA with Dunnett’s test among DH1, *plC*^−^, and *plC*^−^*/plC* cells or among Ax2, *pten*^−^, and *pten*^−^*/pten-GFP* cells]). @, *P* < 0.001 (compared to *plC*^−^ or *pten*^−^ cells [by one-way ANOVA with Dunnett’s test among DH1, *plC*^−^, and *plC*^−^*/plC* cells or among Ax2, *pten*^−^, and *pten*^−^*/pten-GFP* cells]).

### Polyphosphate upregulates inositol 1,4,5-trisphosphate.

**FIG 3:**
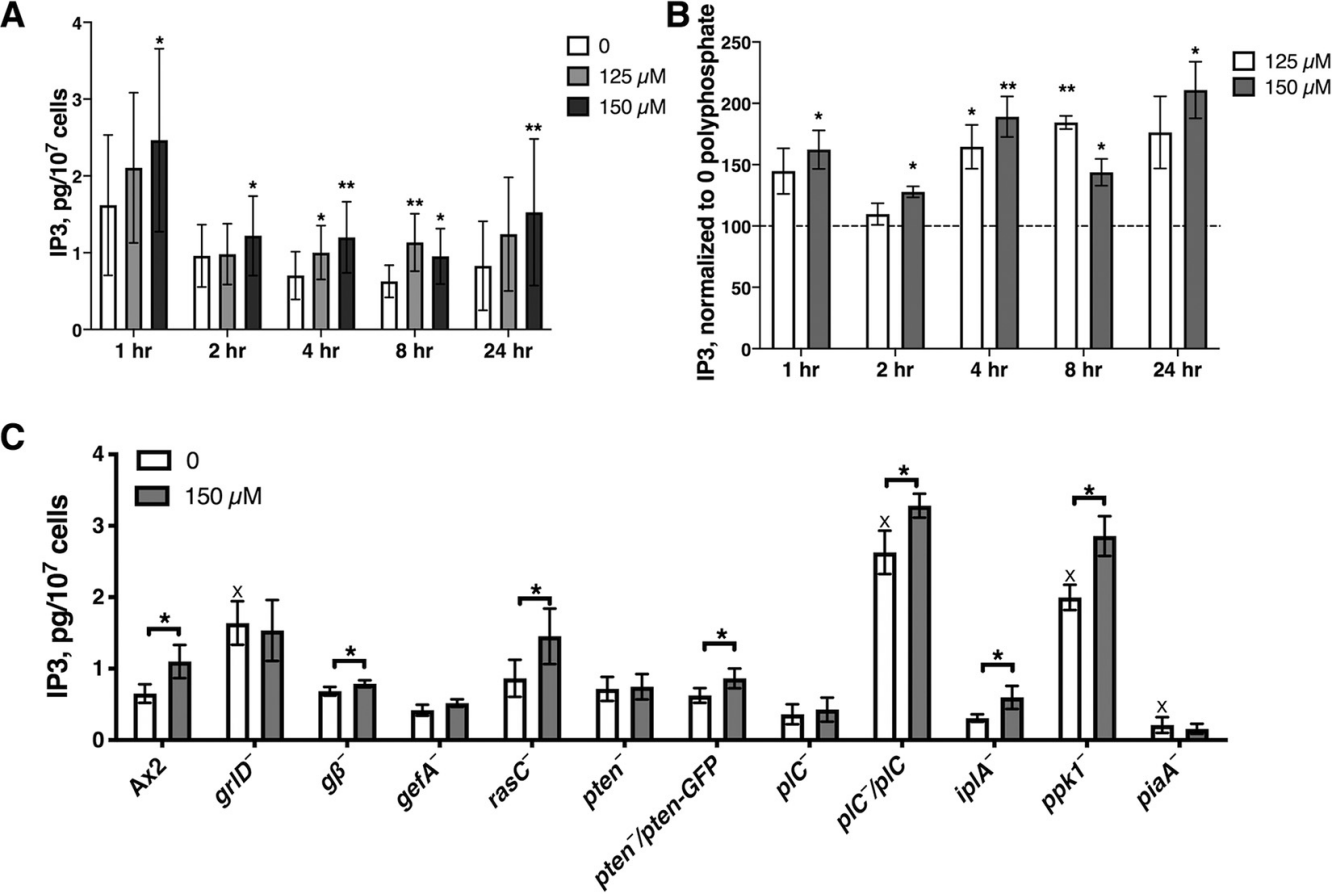
Polyphosphate upregulates inositol 1,4,5-trisphosphate (IP3) levels. (A) Cells were cultured with 0, 125, or 150 μM polyphosphate in 25% HL5 for 1, 2, 4, 8, or 24 h and collected by centrifugation, and IP3 in the cells was measured. (B) For each assay, values were normalized to zero polyphosphate. (C) The indicated cell lines were assayed at 4 h as described above for panel B. All values are means ± SEM (*n* ≥ 4 independent experiments for Ax2 and *n* ≥ 3 for mutants). *, *P* < 0.05; **, *P* < 0.01 (by a two-tailed paired *t* test). X indicates a *P* value of <0.05 compared to Ax2 with no added polyphosphate (by a two-tailed *t* test).

PLC catalyzes the hydrolysis of PIP2 to diacylglycerol (DAG) and inositol 1,4,5-trisphosphate (IP3) (50, 67). PLC and the putative IP3 receptor IplA potentiate polyphosphate inhibition of cell proliferation, suggesting that IP3 might mediate polyphosphate proliferation inhibition. To examine this, we measured the effect of polyphosphate on IP3 levels with an IP3 enzyme-linked immunosorbent assay (ELISA) kit. IP3 levels in Ax2 cells were increased with 125 μM polyphosphate at 4 and 8 h and were increased with 150 μM polyphosphate at 1, 2, 4, 8, and 24 h (Fig. 3A and B). At 4 h, 150 μM polyphosphate increased IP3 in *g*β^−^, *rasC*^−^, *iplA*^−^, and *ppk1*^−^ cells (Fig. 3C). The upregulation of IP3 for *g*β^−^ cells is slight but statistically significant. Polyphosphate did not significantly affect IP3 levels in *grlD*^−^, *gefA*^−^, *pten*^−^, *plC*^−^, and *piaA*^−^ cells, and expressing PTEN in *pten*^−^ cells and PLC in *plC*^−^ cells partially rescued the response (Fig. 3C), possibly because the complementation, with the expression of the cDNA from an actin promoter, causes abnormally high or low levels of the complementing mRNA. Compared to Ax2 cells, the baseline IP3 levels of *grlD*^−^, *plC*^−^*/plC*, and *ppk1*^−^ cells were significantly higher, and the baseline IP3 level of *piaA*^−^ was significantly lower (Fig. 3C). These results indicate that polyphosphate upregulates IP3 in D. discoideum; that this upregulation requires GrlD, GefA, PTEN, PLC, and PiaA; and that Gβ, RasC, IplA, or Ppk1 is dispensable for polyphosphate-induced upregulation of IP3.

### Polyphosphate upregulates cytosolic free Ca^2+^.

**FIG 4:**
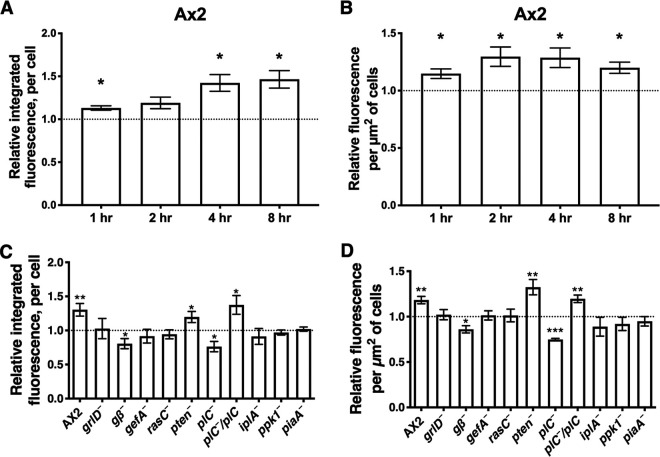
Polyphosphate upregulates cytosolic Ca^2+^. Cells were loaded with the Ca^2+^ detector dye BAPTA-1 dextran and allowed to recover. These cells were then cultured with 0 or 150 μM polyphosphate in 25% HL5 for 1, 2, 4, or 8 h. Calcium levels were measured by microscopy, examining >30 cells per sample. (A and B) Ratios of the fluorescence intensity with 150 μM polyphosphate to the intensity with no polyphosphate. (A) Integrated fluorescence ratio; (B) fluorescence ratio per square micrometer in cell images. (C and D) The indicated cell lines were assayed at 4 h as described above for panels A and B. All values are means ± SEM (*n* ≥ 3 independent experiments). *, *P* < 0.05; **, *P* < 0.01; ***, *P* < 0.001 (compared to no polyphosphate [by a two-tailed *t* test]).

IP3 activates IP3 receptors on the endoplasmic reticulum, leading to Ca^2+^ release from the endoplasmic reticulum lumen to the cytosol in many organisms (50). In D. discoideum, the putative IP3 receptor IplA is localized mostly in cytoplasmic organelles and at very low levels at the plasma membrane and is involved in Ca^2+^ entry into the cytosol in response to chemoattractants (48, 68). As a partial test of the hypothesis that the GrlD-PLC-IP3-IplA-Ca^2+^ pathway is required for the inhibition of proliferation by polyphosphate, we examined the effect of polyphosphate on cytosolic Ca^2+^. 1,2-Bis(2-aminophenoxy)ethane-N,N,N′,N′-tetraacetic acid (BAPTA-1) dextran, which shows increased fluorescence in the presence of Ca^2+^ (69), was loaded into *Dictyostelium* cells by electroporation. This technique loads BAPTA-dextran into the cytosol (69, 70). The BAPTA-1 dextran-loaded cells were then incubated with or without polyphosphate, and Ca^2+^ levels were analyzed based on the total fluorescence per cell (representing the total Ca^2+^ amount) (Fig. 4A and C) and the mean fluorescence per square micrometer of cells (Fig. 4B and D) to exclude the impact of cell size/surface area. By both measurements, polyphosphate increased cytosolic free Ca^2+^ in Ax2 cells (Fig. 4 and Fig. S3). The polyphosphate-induced Ca^2+^ increase happened in 1 h and was maintained for at least 8 h (Fig. 4A and B). These data suggest that polyphosphate upregulates the resting Ca^2+^ level of cells.

10.1128/mBio.01347-21.3 FIG S3 Polyphosphate upregulates cytosolic Ca^2+^. Ax2 cells were loaded with the Ca^2+^ detector dye BAPTA-1 dextran and allowed to recover. These cells were then cultured with 0 or 150 μM polyphosphate for 4 h in 25% HL5 and imaged with a 40× objective on a Ti2 Eclipse inverted epifluorescence microscope (Nikon). Images are representative of results from 3 independent experiments. Bars, 10 μm. Download FIG S3, TIF file, 0.4 MB. Copyright © 2021 Tang et al. 2021 Tang et al. https://creativecommons.org/licenses/by/4.0/ This content is distributed under the terms of the Creative Commons Attribution 4.0 International license.

To test if GrlD, Gβ, GefA, RasC, PTEN, PLC, IplA, Ppk1, and PiaA affect the polyphosphate-induced Ca^2+^ increase, we measured the Ca^2+^ levels of the related mutant cells with or without polyphosphate for 4 h. Polyphosphate did not significantly affect cytosolic free Ca^2+^ in cells lacking GrlD, GefA, RasC, IplA, Ppk1, or PiaA (Fig. 4C and D); increased Ca^2+^ in cells lacking PTEN; and reduced Ca^2+^ in cells lacking Gβ or PLC (Fig. 4C and D). Expressing PLC in *plC*^−^ cells rescued the response to polyphosphate (Fig. 4C and D). Overall, these data suggest that polyphosphate upregulates cytosolic free Ca^2+^ of D. discoideum, and this requires GrlD, Gβ, GefA, RasC, PLC, IplA, Ppk1, and PiaA.

### Polyphosphate inhibits cytokinesis.

Polyphosphate inhibits the proliferation of cells by inhibiting cytokinesis, causing an increased number of multinucleated cells (9). To determine if the signal transduction components identified above are needed for the effect of polyphosphate on cytokinesis, we measured the number of nuclei per cell in the presence or absence of polyphosphate. For wild-type cells (Ax2, Ax3, KAx3, Ax4, DH1, and JH10), polyphosphate increased the number of nuclei per cell (Table 3). This effect was not observed in cells lacking GrlD, Gβ, RasC, PTEN, PLC, IplA, Ppk1, and PiaA (Table 3). Expressing PTEN in *pten*^−^ cells and PLC in *plC*^−^ cells rescued or partially rescued the sensitivity to polyphosphate (Table 3). These data suggest that most of the potential signaling components identified above are needed for polyphosphate inhibition of cytokinesis.

**TABLE 3:** The potential polyphosphate pathway components are needed for polyphosphate induced cell multinucleation^*a*^

Cell type	Polyphosphate concn (μM)	Mean no. of nuclei/100 cells ± SEM	Mean % of cells with no. of nuclei ± SEM		
			1	2	3+
Ax2	0	108 ± 2	93.2 ± 1.5	6.5 ± 1.3	0.3 ± 0.2
	150	123 ± 3**	79.4 ± 2.1***	18.7 ± 1.5***	1.9 ± 0.8

Ax3	0	114 ± 2	88.5 ± 1.5	10.0 ± 1.0	1.5 ± 0.6
	150	138 ± 5**	71.7 ± 2.6***	21.5 ± 1.3***	6.8 ± 1.7*

KAx3	0	103 ± 2	97.0 ± 1.3	2.7 ± 1.1	0.3 ± 0.3
	150	127 ± 3**	75.2 ± 2.6***	23.1 ± 2.6***	1.7 ± 0.9

Ax4	0	102 ± 1	98.3 ± 0.6	1.6 ± 0.6	0.2 ± 0.2
	150	131 ± 4***	75.0 ± 3.1***	20.8 ± 3.2***	4.2 ± 0.8*

DH1	0	118 ± 2	84.3 ± 1.1	12.8 ± 1.0	2.6 ± 1.0
	150	135 ± 3**	69. 2 ± 2.2***	27.1 ± 2.1***	3.7 ± 0.5

JH10	0	124 ± 4	82.1 ± 2.4	14.5 ± 2.0	3.5 ± 0.6
	150	147 ± 5*	64.5 ± 4.2***	27.5 ± 3.5**	8.0 ± 1.0*

*grlD* ^−^	0	104 ± 1	96.2 ± 1.2	3.8 ± 1.2	0 ± 0
	150	105 ± 2	95.3 ± 1.4	4.7 ± 1.2	0 ± 0

*g*β^−^	0	101 ± 1	98.9 ± 0.3	1.1 ± 0.3	0 ± 0
	150	102 ± 1	98.3 ± 0.7	1.4 ± 0.6	0 ± 0

*gefA* ^−^	0	109 ± 2	91.2 ± 1.6	8.5 ± 1.5	0.3 ± 0.3
	150	120 ± 4*	81.0 ± 3.1*	17.9 ± 2.8*	1.1 ± 0.4*

*rasC* ^−^	0	116 ± 4	86.8 ± 2.2	11.3 ± 1.5	1.9 ± 0.7
	150	117 ± 5	85.9 ± 3.0	12.5 ± 2.5	1.6 ± 0.5

*pten* ^−^	0	120 ± 2	82.4 ± 1.9	16.0 ± 1.9	1.6 ± 0.1
	150	116 ± 4	86.4 ± 2.6	12.1 ± 2.0	1.5 ± 0.7

*pten* ^−^ */pten-GFP*	0	114 ± 6	87.2 ± 4.2	12.1 ± 3.9	0.7 ± 0.3
	150	123 ± 5	79.9 ± 3.2***	17.7 ± 2.9	2.3 ± 0.3*

*plC* ^−^	0	106 ± 3	94.1 ± 2.5	5.9 ± 2.5	0 ± 0
	150	110 ± 4	89.7 ± 3.0	10.3 ± 3.0	0 ± 0

*plC* ^−^ */plC*	0	106 ± 1	94.6 ± 1.0	5.3 ± 0.9	0.2 ± 0.2
	150	121 ± 4*	81.4 ± 2.6**	16.6 ± 2.0**	2.0 ± 0.7**

*iplA* ^−^	0	103 ± 1	97.3 ± 0.5	2.7 ± 0.5	0 ± 0
	150	104 ± 1	96.5 ± 1.0	3.5 ± 1.0	0 ± 0

*ppk1* ^−^	0	106 ± 2	94.9 ± 0.9	4.7 ± 0.8	0.4 ± 0.2
	150	111 ± 2	90.6 ± 1.8	8.5 ± 2	0.9 ± 0.4

*piaA* ^−^	0	102 ± 1	98.4 ± 0.5	1.6 ± 0.5	0 ± 0
	150	104 ± 1	96.0 ± 1.0	4.0 ± 1.0	0 ± 0

a The number of nuclei and percentage of cells with 1, 2, and 3 or more nuclei were measured by counts of DAPI (4′,6-diamidino-2-phenylindole)-stained cells. Cells were examined by using an epifluorescence microscope with a 40× lens, and for each condition, at least 100 cells were counted. Values are means ± SEM (*n* ≥ 3 independent experiments). *, *P* < 0.05; **, *P* < 0.01; ***, *P* < 0.001 (compared to no polyphosphate [by a two-tailed *t* test]).

### Polyphosphate does not upregulate total Ras activity.

Ras is activated when it binds to GTP and inactivated when it binds to GDP (71). As RasC is required for the polyphosphate effect on proliferation, we hypothesized that polyphosphate might affect RasC activation. Due to the lack of a RasC-specific detection method, we tested the effect of polyphosphate on the total Ras activity of Ax2 cells. There are 11 Ras proteins in *Dictyostelium* (72). We did not observe any significant difference in active-Ras levels between cells cultured with 0 and those cultured with 150 μM polyphosphate for 1, 4, and 24 h (Fig. S4). This suggests that the RasC activity needed for the polyphosphate proliferation inhibition pathway might be only a small fraction of the total Ras activity.

10.1128/mBio.01347-21.4 FIG S4 Polyphosphate does not upregulate total Ras activity. Ax2 cells were cultured with 0 or 150 μM polyphosphate for 1, 4, or 24 h in 25% HL5. At the indicated times, cells were lysed, and the active Ras levels in the lysates were measured. The active Ras levels were normalized to the value for the no-polyphosphate-added group at each time point. Values are means ± SEM (*n* = 3 independent experiments). Download FIG S4, TIF file, 2 MB. Copyright © 2021 Tang et al. 2021 Tang et al. https://creativecommons.org/licenses/by/4.0/ This content is distributed under the terms of the Creative Commons Attribution 4.0 International license.

## DISCUSSION

**FIG 5:**
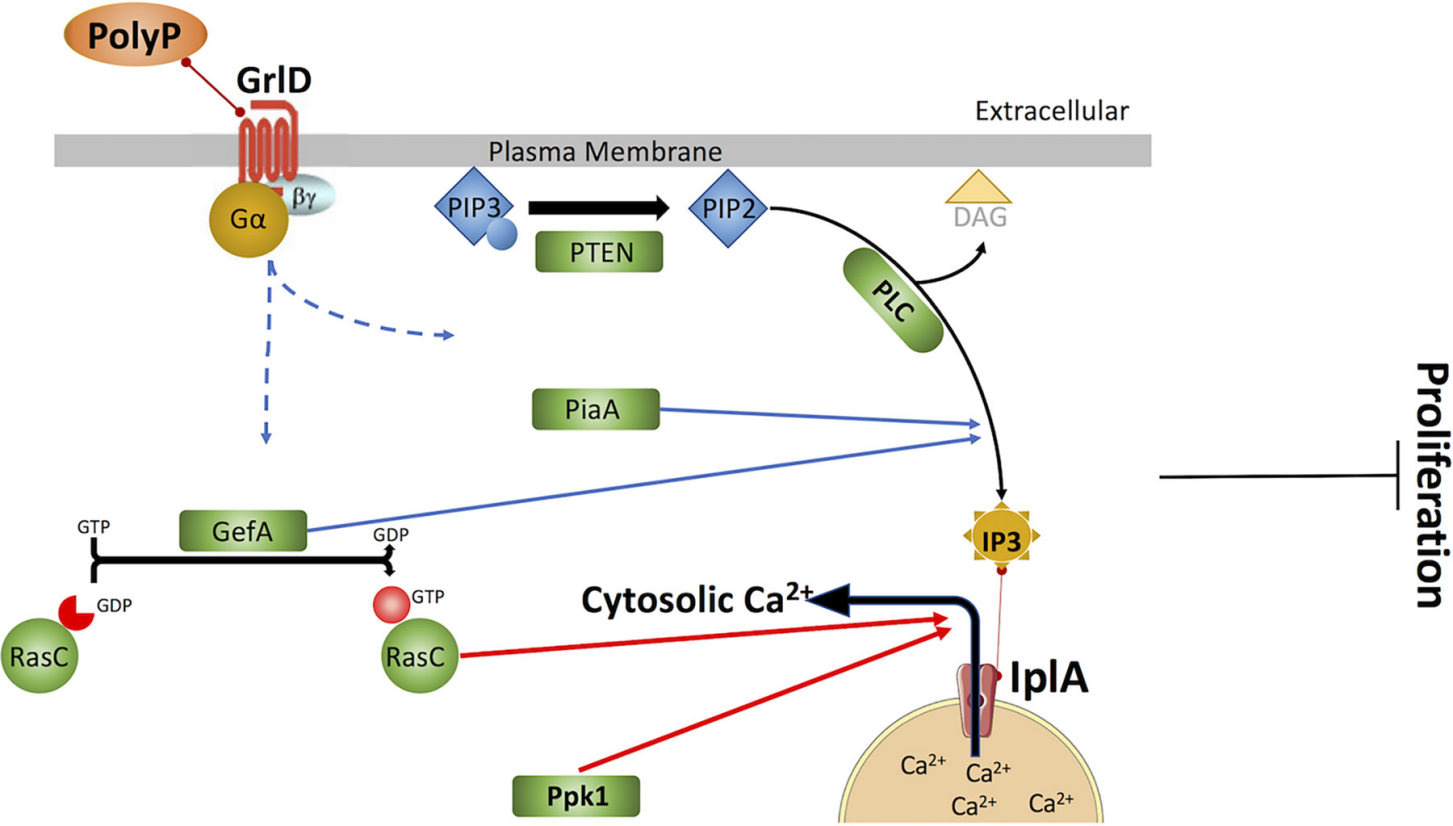
Hypothesized signaling pathway of polyphosphate inhibition of cell proliferation. Polyphosphate binds to the GrlD receptor, and the polyphosphate signal is transmitted through Gαs and Gβγ. Downstream, PTEN catalyzes the conversion of PIP3 to PIP2, and PLC catalyzes the hydrolysis of PIP2 to IP3 and DAG; IP3 binds to the putative IP3 receptor IplA, releasing Ca^2+^ to the cytosol. Polyphosphate upregulates IP3 levels and cytosolic Ca^2+^ levels through PTEN, PLC, and IplA. GefA catalyzes the conversion of GDP-bound RasC to GTP-bound RasC. GefA and PiaA are required for polyphosphate to upregulate IP3, and RasC and Ppk1 are required for polyphosphate to upregulate cytosolic Ca^2+^. The intermediate components between PiaA and IP3, GefA and IP3, RasC and Ca^2+^, and Ppk1 and Ca^2+^ are unknown. Together, these components mediate polyphosphate inhibition of cell proliferation.

We screened 52 signal transduction pathway mutants for sensitivity to polyphosphate-induced proliferation inhibition. We found that in addition to the previously reported GrlD receptor and RasC (17), Gβ, GefA, PakD, PlaA, PTEN, PLC, IplA, Dd5p4, Erk1, MekA, I6kA, Ppk1, Gdt1, Gdt2, Gdt4, PiaA, and PKA-C potentiate polyphosphate inhibition of cell proliferation, suggesting that a complex signal transduction pathway mediates this example of an autocrine proliferation inhibition mechanism (Fig. 5). Compared to their respective parental cells, *g*β^−^, *gefA*^−^, *rasC*^−^, *pten*^−^, *plC*^−^, *iplA*^−^, *ppk1*^−^, and *piaA*^−^ cells showed strongly reduced sensitivity to polyphosphate proliferation inhibition but not as abolished as that of *grlD*^−^ cells. This suggests that there might be branched pathways downstream of the receptor GrlD. We observed that the lack of any tested Gα subunit did not abolish the cells’ sensitivity to polyphosphate inhibition of cell proliferation (Fig. 1B). This is possibly because multiple Gα subunits are involved in the polyphosphate pathway, and the loss of a single Gα could be compensated for by other Gα subunits, or the Gα subunit(s) activated by polyphosphate is among the untested Gα subunits. Many mutants with abnormal proliferation (see Table S2 in the supplemental material) do not appear to be part of the polyphosphate signal transduction pathway, indicating that, as expected, many other factors besides polyphosphate affect proliferation.

The polyphosphate signal transduction pathway appears to use components that regulate proliferation in other systems. Ras-, PLC-, and IP3-induced Ca^2+^ release promotes proliferation, and PTEN and PKA inhibit proliferation in mammalian systems (73–79). Inhibition of Ras-, PLC-, or IP3-induced Ca^2+^ release inhibits cell proliferation in various cell types (75, 80–82). The overexpression of PTEN inhibits cell proliferation in many cancer cell lines (76, 77, 83), and the activation of PKA inhibits vascular smooth cell proliferation induced by injury (78, 79).

Consistent with the observation that polyphosphate induces Erk phosphorylation (17), we found that cells lacking Erk1 showed reduced sensitivity to polyphosphate. Polyphosphate-induced Erk phosphorylation requires RasC (17). Combined with the data in this report, this suggests that RasC-Erk1 is part of a pathway involved in polyphosphate proliferation inhibition.

Polyphosphate is a prestarvation factor that primes *Dictyostelium* cells for development (17). Polyphosphate induces the expression of the early-onset development protein CsaA (17). Cells lacking the polyphosphate receptor GrlD showed an impaired response to the starvation-induced expression of the aggregation markers CsaA, Car1 (cyclic AMP receptor 1), and AcaA (adenylyl cyclase A) and could not perform normal development (23). Many signal transduction pathway components affecting the *Dictyostelium* growth-development transition also affect polyphosphate inhibition of proliferation (Fig. 1G). As starvation causes both the cessation of proliferation and the initiation of development, many components involved in the initiation of development might also affect the proliferation inhibition response. Inducing these development-related components could be part of the mechanism whereby high concentrations of extracellular polyphosphate allow cells to anticipate starvation. How bacteria, by either consuming the polyphosphate secreted by *Dictyostelium* cells or secreting their own polyphosphate, interfere with *Dictyostelium* polyphosphate signaling is unclear. An intriguing observation is that *Dictyostelium* cells can proliferate on lawns of Pseudomonas aeruginosa bacteria that lack the bacterial polyphosphate kinase PPK1 but not on lawns of wild-type P. aeruginosa cells (84). One possibility for this result is that the polyphosphate from wild-type P. aeruginosa cells causes *Dictyostelium* cells to stop proliferating.

Many components of the AprA and cAMP signal transduction pathways (some components, such as the cAMP receptor cAR1 [85], were not examined) did not affect polyphosphate inhibition of cell proliferation. For those components in these two pathways that potentiated polyphosphate-induced proliferation inhibition, the effect on polyphosphate inhibition was relatively mild. PiaA and Lst8 are both Tor complex 2 components (60), but *piaA*^−^ cells showed some impairment of polyphosphate signaling, while *lst8*^−^ cells showed no significant inhibition, suggesting that PiaA and Lst8 have independent functions.

We tested the effect of the mutants that attenuate polyphosphate-mediated inhibition of proliferation in a shaking culture. *pten*^−^ and *ppk1*^−^ cells proliferated abnormally slowly and *g*β^−^ (66) and *plC*^−^ cells proliferated abnormally quickly at low cell densities, and *iplA*^−^, *ppk1*^−^, and *piaA*^−^ cells proliferated abnormally quickly at high cell densities. The maximal cell densities of *g*β^−^, *gefA*^−^, *iplA*^−^, *ppk1*^−^, and *piaA*^−^ cells were abnormally high, and those of *pten*^−^ and *plC*^−^ cells were abnormally low. Compared to wild-type cells, we expected that mutant cells with reduced sensitivity to polyphosphate would proliferate faster and reach higher maximal cell densities. However, *pten*^−^ cells proliferated slower, and *pten*^−^ and *plC*^−^ cells had a lower maximal cell density. The accumulated extracellular polyphosphate levels of these four mutants might be abnormally high, or the genes knocked out could be required for regulating proliferation through other pathways. Compared to DH1 or *plC*^−^ cells, *plC*^−^*/plC* cells (overexpressing PLC in *plC*^−^ cells) proliferate faster and reach a higher maximal density (Table 2), suggesting that the overexpression of PLC does more than just restore the function of the lost gene. As the concentration of accumulated extracellular polyphosphate is low when the cell density is low (9), the faster-proliferation phenotype at this stage supports the hypothesis that *plC* regulates proliferation through pathways other than polyphosphate. The lack, or overexpression, of PLC caused a faster-proliferation phenotype, indicating that the PLC effect on cell proliferation is dependent on PLC levels.

Polyphosphate proliferation inhibition is potentiated by proteins in the PLC/IP3 pathway. We found that polyphosphate upregulates cellular IP3 levels; that this requires GrlD, GefA, PTEN, PLC, and PiaA; and that Gβ, RasC, IplA, and Ppk1 are not required for polyphosphate to upregulate IP3. Together, these results suggest that polyphosphate activates a signal transduction pathway that upregulates IP3 levels. IP3 activates IP3 receptors on the endoplasmic reticulum, leading to Ca^2+^ release from the endoplasmic reticulum lumen to the cytosol in many organisms (50). We found that polyphosphate upregulates cytosolic Ca^2+^ levels and that this requires GrlD, Gβ, GefA, RasC, PLC, IplA, Ppk1, and PiaA. Polyphosphate thus appears to upregulate the resting cytosolic Ca^2+^ of *Dictyostelium* cells, similar to the effects of other signals on resting cytosolic Ca^2+^ in other systems (86, 87).

Polyphosphate upregulated both IP3 levels and cytosolic Ca^2+^ levels of Ax2 cells but did not significantly alter either IP3 levels or cytosolic Ca^2+^ levels of *grlD*^−^, *gefA*^−^, and *piaA*^−^ cells. These results suggest that GrlD, GefA, and PiaA function upstream of elevating IP3 in the polyphosphate pathway (Fig. 5). As expected, polyphosphate upregulated IP3 levels and did not alter cytosolic Ca^2+^ levels in cells lacking the inositol 1,4,5-trisphosphate receptor-like protein IplA. In cells lacking RasC or Ppk1, polyphosphate upregulated IP3 but did not affect cytosolic Ca^2+^. In cells lacking Gβ, polyphosphate upregulated IP3 but downregulated cytosolic Ca^2+^. A possible explanation is that Gβ, RasC, and Ppk1 are required for IP3 to activate the IplA receptor to release Ca^2+^ to the cytosol and that GrlD might use components in addition to G proteins to transduce extracellular signals. Unexpectedly, the IP3 levels of cells lacking PTEN or PLC were not altered by polyphosphate, but the cytosolic Ca^2+^ of cells lacking PTEN or PLC was upregulated or downregulated, respectively. These results suggest that polyphosphate can regulate cytosolic Ca^2+^ levels through a pathway not involving IP3.

Ppk1 mediates intracellular polyphosphate production, and the intracellular polyphosphate of *ppk1*^−^ cells is undetectable (12). How intracellular (as opposed to extracellular) polyphosphate or Ppk1 affects extracellular polyphosphate-induced proliferation inhibition is unclear. As polyphosphate can bind to free divalent cations such as Ca^2+^ and Mg^2+^ (10), one hypothesis is that intracellular polyphosphate might bind to the extracellular polyphosphate-induced elevated cytosolic free Ca^2+^, and the intracellular polyphosphate-Ca^2+^ complex could then function as a second messenger. If this is the case, compared to Ax2 cells, cells lacking Ppk1 should show a higher increase of the fluorescence signal with the BAPTA-1 dextran method after stimulating cells with polyphosphate, as polyphosphate-bound Ca^2+^ could not be detected by BAPTA-1. However, cells lacking Ppk1 lost the polyphosphate-induced cytosolic free Ca^2+^ increase (Fig. 4) while still showing a polyphosphate-induced IP3 increase (Fig. 3). This result disproves the hypothesis of a polyphosphate-Ca^2+^ elevation and a Ca^2+^-bound polyphosphate pathway. This indicates that Ppk1/intracellular polyphosphate functions downstream of IP3 and upstream of Ca^2+^ elevation.

Besides proliferation inhibition, polyphosphate inhibits proteasome activity, promotes aggregation, and regulates actin polymerization in D. discoideum cells (23). In both 25% and 100% HL5, polyphosphate reduces proteasome activity, and this requires GrlD and RasC (23). However, in 25% HL5 but not 100% HL5, MG132-induced inhibition of proteasome activity inhibits proliferation (23). In human colon cancer HCT116 cells, the proteasome inhibitor MG132 increases intracellular Ca^2+^ levels (88), and in mouse embryonic fibroblasts, chelating calcium by BAPTA-acetoxymethyl ester (AM) decreases proteasome activity, while increasing intracellular Ca^2+^ with 2 mM extracellular Ca^2+^ and ionomycin treatment increases proteasome activity (89). In *Dictyostelium*, whether there is cross talk in the polyphosphate signal transduction pathway between proteasome activity and IP3/Ca^2+^ levels is unclear.

In this report, we identified 7 signaling components in the polyphosphate pathway and showed that polyphosphate appears to inhibit *Dictyostelium* proliferation through pathways including the IP3/Ca^2+^ pathway. An intriguing possibility is that similar mechanisms may be used in other eukaryotes for autocrine proliferation inhibition and group and tissue size regulation.

## MATERIALS AND METHODS

### Cell culture and strains.

Dictyostelium discoideum strains were obtained from the *Dictyostelium* stock center (90) and were parental/wild-type strains Ax2 (Dictybase identifier DBS0237699) (91), Ax3 (DBS0235542) (92), KAx3 (DBS0266758) (93), Ax4 (DBS0302402) (94), DH1 (DBS0235700) (85), JH8 (DBS0236454) (95), JH10 (DBS0236449) (95), and HPS400 (DBS0236312) (96); mutants *grlD*^−^ (DBS0350227) (23), *rasC*^−^ (DBS0236853) (24), *gefA*^−^ (DBS0236896) (25), *rasG*^−^ (DBS0236862) (97), *g*β^−^ (DBS0236531) (26), *g*α*1*^−^ (DBS0236088) (27), *g*α*2*^−^ (DBS0236575) (37), *g*α*3*^−^ (DBS0235986) (98), *g*α*4*^−^ (DBS0235984) (99), *g*α*5*^−^ (DBS0236451) (100), *g*α*7*^−^ (DBS0236106) (101), *g*α*8*^−^ (DBS0236107) (101), *g*α*9*^−^ (DBS0236109) (102), *aprA*^−^ (DBS0235509) (28), *cfaD*^−^ (DBS0302444) (30), *pakD*^−^ (DBS0350281) (29), *rblA*^−^ (DBS0236877) (31), *cnrN*^−^ (DBS0302655) (32), *qkgA*^−^ (DBS0236839) (35), *bzpN*^−^ (DBS0349965) (36), *scrA*^−^ (DBS0236926) (40), *elmoE*^−^ (DBS0350065) (41), *gcA*^−^*/sgcA*^−^ (DBS0302679) (42), *racC*^−^ (DBS0350272) (103), *plA2*^−^ (DBS0238068) (39), *pikA*^−^*/pikB*^−^ (DBS0236766) (44), *dagA*^−^ (DBS0235559) (45), *pten*^−^ (DBS0236830) (46), *pten*^−^*/pten-GFP* (DBS0236831) (46), *plC*^−^ (DBS0236793) (47), *plC*^−^*/plC* (DBS0236795) (104), *iplA*^−^ (DBS0236260) (48), *Dd5p4*^−^ (DBS0266692) (49), *erk1*^−^ (DBS0350622) (52), *erk1*^−^*/erk2*^−^ (DBS0351256) (105), *mekA*^−^ (DBS0236541) (53), *smkA*^−^ (DBS0236938) (53), *i6kA*^−^ (DBS0236426) (106), *ppk1*^−^ (DBS0350686) (12), *csaA*^−^ (DBS0236957) (56), *smlA*^−^ (DBS0236939) (58), *piaA*^−^ (DBS0349879) (107), *lst8*^−^ (DBS0236517) (60), *pkaC*^−^ (DBS0236783) (64), *pkcA*^−^ (DBS0350916) (108), *amtA*^−^ (DBS0235497) (57), *sibA*^−^ (DBS0236935) (109), *tpC2*^−^ (65), and *trpp*^−^ (65) (gifts from Pierre Cosson, University of Geneva, Geneva, Switzerland); mutants *mcln*^−^ (DBS0350059) (110) and *wasA*^−^ (gifts from Robert Insall, Beatson Institute for Cancer Research, Glasgow, UK) (43); and mutants *gdt1*^−^*/gdt2*^−^, *gdt2*^−^, and *gdt4*^−^ (gifts from Adam Kuspa, Baylor College of Medicine) (see Table S1 in the supplemental material). As described previously, all mutants were confirmed by PCR (33). Cells were cultured at 21°C in a shaking culture at 175 rpm in HL5 (Formedium Ltd., Norwich, England). Cells were counted by a hemocytometer.

10.1128/mBio.01347-21.5 TABLE S1 *Dictyostelium* cell lines used in this report. The mutant strain, the Dictybase identifier, and the parental strain are listed. Download Table S1, DOCX file, 0.02 MB. Copyright © 2021 Tang et al. 2021 Tang et al. https://creativecommons.org/licenses/by/4.0/ This content is distributed under the terms of the Creative Commons Attribution 4.0 International license.

### Proliferation inhibition and counts of nuclei.

Polyphosphate was prepared by dissolving 0.474 g of ∼46-mer (average length) S0169 sodium polyphosphate (Spectrum, New Brunswick, NJ) in 10 ml of PBM (20 mM KH_2_PO_4_, 0.01 mM CaCl_2_, 1 mM MgCl_2_ [pH 6.1]) (23) to make a 10 mM stock; the final pH was 6.1, and the pH was thus not adjusted. Mid-log-phase cells (1 × 10^6^ to 4 × 10^6^ cells/ml) cultured in HL5 were collected by centrifugation at 1,000 × *g* for 3 min, washed once by resuspension of the cells in PBM and centrifugation at 1,000 × *g* for 3 min, and then resuspended in fresh HL5 to 6 × 10^6^ cells/ml. Cell cultures were started by mixing 100 μl of these cells with 300 μl of PBM or HL5 containing the indicated concentrations (adjusted for the dilution with cells) of polyphosphate in the well of a type 353047 24-well plate (Corning, Corning, NY) and incubated in a humid box for 24 h at 21°C. For work with cells in 25% HL5, HL5 was diluted by mixing 1 volume of HL5 with 3 volumes of PBM. Cells were counted at 24 h, and the cell density normalized to the density with no added polyphosphate was calculated. The doubling time and maximal density of each strain were calculated as described previously (23), and the numbers of nuclei per cell were counted as described previously (28). Curve fits and IC_50_ calculations were done using Prism (GraphPad, San Diego, CA) with nonlinear regression (sigmoidal dose-response, variable slope, and top constrained to 100).

### Extraction and measurement of inositol (1,4,5)-trisphosphate.

Cells were grown to mid-log phase and counted, and ∼2 × 10^7^ cells were collected by centrifugation, washed with PBM as described above, and then resuspended and incubated in 10 ml 25% HL5 (diluted with PBM) with 0 or 150 μM polyphosphate in a shaking culture at 175 rpm. After 1, 2, 4, 8, or 24 h, cells were collected by centrifugation at 1,000 × *g* for 3 min and resuspended in 110 μl of the supernatant from the centrifugation step in 1.7-ml Eppendorf tubes. From the resuspended cells, 10 μl was taken out for cell counts, and the remaining cells were mixed with 100 μl 3.5% perchloric acid and incubated on ice for 15 min as described previously (111). Half-saturated KHCO_3_ (50 μl) was then added to the 200-μl mix to neutralize the lysates, and CO_2_ was allowed to escape. The material was then clarified by centrifugation at 14,000 × *g* for 5 min at 4°C. The supernatant (200 μl) of each tube was transferred to new prechilled 1.7-ml tubes and stored at 0°C. The IP3 levels in the clarified lysates were measured with a type 2515875 IP3 ELISA kit (MyBioSource, San Diego, CA) less than 1 week after extraction. The baseline IP3 levels that we measured (Fig. 3A and C) are far lower than the levels previously reported using an isotope dilution kit that has been discontinued by the manufacturer (picograms versus micrograms per 10^7^ cells) (112, 113). Both kits detect IP3 levels based on a competition binding strategy, but the isotope kit used an IP3 binding protein prepared from bovine adrenal cortex, and the ELISA kit uses an anti-IP3 antibody. We hypothesize that the difference between the measured IP3 levels could be caused by the specificity of the anti-IP3 antibody being much higher than that of the bovine IP3 binding protein.

### Measurement of cytosolic free Ca^2+^.

Mid-log-phase cells (3 × 10^6^) were collected by centrifugation at 1,000 × *g* for 3 min, washed with ice-cold Sorensen’s buffer (14.7 mM KH_2_PO_4_, 2 mM Na_2_HPO_4_ [pH 6.1]) twice (each time collecting cells by centrifugation and resuspension), and then resuspended in 95 μl ice-cold Sorensen’s buffer. As described previously (70), 90 μl of washed cells was then mixed with 10 μl 25 mg/ml BAPTA-1 dextran at a 10,000 molecular weight (MW) (Invitrogen, Eugene, OR), loaded into an EC2L 2-mm electroporation cuvette (Midsci, Valley Park, MO), and pulsed once with 850 V at 10 μF and 200 Ω in a GenePulser XCell electroporator (Bio-Rad, Hercules, CA). The cells were then collected by centrifugation, resuspended in 1 ml HL5, and incubated for 30 min at 21°C in a shaking culture at 175 rpm. The cells were then diluted and incubated at 1 × 10^6^ cells/ml with 150 μM polyphosphate or an equal volume of PBM in 25% HL5 for 0.5, 1.5, 3.5, or 7.5 h. The cells were then diluted to 0.3 × 10^6^ cells/ml with 150 μM polyphosphate or an equal volume of PBM in 25% HL5, and 300 μl of diluted cells was allowed to adhere in the well of a type 94.6190.802 8-well tissue culture chamber (Sarstedt, Nümbrecht, Germany) for 30 min. Cells were imaged with a 40× objective on a Ti2 Eclipse inverted epifluorescence microscope (Nikon, Melville, NY). The fluorescence intensity was analyzed by using ImageJ.

### Measurement of active Ras.

Cells were grown to mid-log phase (1 × 10^6^ to 4 × 10^6^ cells/ml) and counted, and 1 × 10^6^ cells were collected by centrifugation, washed with PBM as described above, and then resuspended and incubated in 1 ml 25% HL5 (diluted with PBM) with 0 or 150 μM polyphosphate. After 1, 4, or 24 h, cells were lysed, and the active Ras levels in the lysates were measured with a Ras activation assay kit (Cytoskeleton, Denver, CO). All the procedures were performed according to the manufacturer’s manual except that the cell lysate with 30 μg protein was mixed with 30 μg Raf-RBD protein beads for active Ras pulldown.

### Statistics.

Statistical analyses were done using Prism (GraphPad). Significance was defined as a *P* value of <0.05.
