# Supplementary material for: An Autocrine Negative Feedback Loop Inhibits Dictyostelium discoideum Proliferation through Pathways Including IP3/Ca2+
Source: mBio. 2021 Jun 22;12(3):e01347-21. doi: 10.1128/mBio.01347-21 (PMC8262924; doi:10.1128/mBio.01347-21)
Supplement: TABLE S2 [file mbio.01347-21-st002.docx]

**Table S2.** **Some mutants have abnormal proliferation in 25% HL5.** The indicated cell lines were tested for proliferation for 24 hours. Cells were cultured in 25% HL5, starting with 1.5 x 10^6^ cells/ml. All values are mean ± SEM, n ≥ 3 independent experiments. *, p < 0.05, **, p < 0.01, ***, p < 0.001 (two-tailed t-test compared to parental cell line).

|  | Cell Density at 24 hr, 10^6^ cells/ml | | Doubling Time, hours | |
| --- | --- | --- | --- | --- |
| Ax2 | 3.9 ± 0.1 |  | 17.7 ± 0.7 |  |
| Ax3 | 6.7 ± 0.1 |  | 11.1 ± 0.1 |  |
| KAx3 | 2.3 ± 0.1 |  | 39.1 ± 2.1 |  |
| Ax4 | 5.6 ± 0.6 |  | 12.9 ± 1.1 |  |
| DH1 | 4.6 ± 0.2 |  | 15.0 ± 0.7 |  |
| JH8 | 3.3 ± 0.3 |  | 21.6 ± 2.1 |  |
| JH10 | 4.8 ± 0.3 |  | 14.5 ± 0.7 |  |
| HPS400 | 3.2 ± 0.1 |  | 22.3 ± 1.0 |  |
| *grlD¯* | 3.3 ± 0.8 | * | 20.8 ± 0.9 | * |
| *rasC¯* | 5.7 ± 0.5 | * | 13.2 ± 1.5 | * |
| *gefA¯* | 5.6 ± 0.3 |  | 12.8 ± 0.9 |  |
| *gβ¯* | 4.1 ± 0.3 |  | 17.1 ± 0.9 |  |
| *gα1¯* | 6.4 ± 0.4 | *** | 11.6 ± 0.4 | *** |
| *gα2¯* | 3.5 ± 0.3 | * | 22 ± 2.6 | * |
| *gα3¯* | 5.2 ± 0.5 | ** | 13.6 ± 0.8 | *** |
| *gα4¯* | 4.7 ± 0.6 |  | 15.3 ± 1.6 |  |
| *gα5¯* | 4.6 ± 0.4 |  | 16 ± 2.2 |  |
| *gα7¯* | 3 ± 0.2 | * | 26.9 ± 4.9 |  |
| *gα8¯* | 3.4 ± 0.5 | * | 21.1 ± 1.7 |  |
| *gα9¯* | 5.5 ± 0.4 |  | 13.1 ± 0.8 | * |
| *aprA¯* | 5 ± 0.4 | * | 14.1 ± 0.9 |  |
| *cfaD¯* | 5.8 ± 0.2 | *** | 12.5 ± 0.7 | *** |
| *pakD¯* | 3.8 ± 0.5 |  | 18 ± 0.9 |  |
| *rblA¯* | 5.7 ± 1.0 | ** | 12.6 ± 0.7 | *** |
| *cnrN¯* | 5.3 ± 0.5 |  | 16 ± 3.3 |  |
| *qkgA¯* | 6.7 ± 0.6 | *** | 11.3 ± 0.5 | *** |
| *bzpN¯* | 4.8 ± 0.4 |  | 15.1 ± 1.7 |  |
| *scrA¯* | 4.3 ± 0.5 |  | 16.6 ± 1.4 |  |
| *elmoE¯* | 4.7 ± 0.1 |  | 14.9 ± 1.3 |  |
| *gcA¯/sgcA¯* | 3.9 ± 0.3 | *** | 17.4 ± 0.5 | *** |
| *wasA¯* | 7.3 ± 0.2 |  | 10.5 ± 0.2 |  |
| *racC¯* | 3.5 ± 0.3 |  | 20.1 ± 1.3 |  |
| *plaA¯* | 4.9 ± 0.2 | ** | 14.3 ± 0.7 | * |
| *pikA¯/pikB¯* | 3.0 ± 0.1 | * | 24 ± 1.8 | ** |
| *dagA¯* | 4.5 ± 0.1 | *** | 15.3 ± 0.3 | *** |
| *pten¯* | 3.9 ± 0.3 |  | 17.5 ± 0.5 |  |
| *pten¯/pten-GFP* | 3.1 ± 0.1 | ** | 22.6 ± 1.0 | ** |
| *plC¯* | 5.2 ± 0.3 |  | 13.8 ± 0.7 |  |
| *plC¯/plC* | 6.1 ± 0.3 | ** | 11.9 ± 0.5 | ** |
| *iplA¯* | 4.4 ± 0.5 |  | 15.8 ± 0.9 |  |
| *Dd5P4¯* | 3.4 ± 0.3 |  | 22.8 ± 4.4 |  |
| *erk1¯* | 5.3 ± 0.6 | *** | 13.3 ± 0.5 | *** |
| *erk1¯/ekr2¯* | 3.9 ± 0.3 |  | 19.5 ± 3.2 |  |
| *mekA¯* | 3.7 ± 0.2 |  | 19.1 ± 1.8 |  |
| *smkA¯* | 4.9 ± 0.3 | *** | 14.3 ± 0.8 | * |
| *i6kA¯* | 5.9 ± 0.4 | *** | 12.3 ± 0.5 | *** |
| *ppk1¯* | 4.6 ± 0.5 |  | 15.5 ± 1.2 |  |
| *gdt1¯/gdt2¯* | 3.8 ± 0.5 |  | 20.1 ± 2.5 |  |
| *gdt2¯* | 4 ± 0.3 | * | 17.5 ± 1.2 | * |
| *gdt4¯* | 4.2 ± 0.3 |  | 16.9 ± 0.4 | * |
| *csaA¯* | 4.2 ± 0 |  | 16.5 ± 1.4 |  |
| *smlA¯* | 4 ± 0.3 |  | 16.9 ± 0.2 |  |
| *amtA¯* | 4.2 ± 0.2 | * | 16.8 ± 1.4 |  |
| *piaA¯* | 2 ± 0.4 | *** | 79.4 ± 22.2 | * |
| *lst8¯* | 3.1 ± 0.2 |  | 25.1 ± 4.9 |  |
| *pkaC¯* | 3.6 ± 0.2 |  | 19.4 ± 1.3 | * |
| *pkcA¯* | 3.2 ± 0.4 | * | 22 ± 1.8 | * |
| *sibA¯* | 4.1 ± 0.3 |  | 16.9 ± 0.9 |  |
| *tpC2¯* | 4.1 ± 0.5 |  | 16.9 ± 1.2 |  |
| *trpp¯* | 4.8 ± 0.4 |  | 14.8 ± 1.4 |  |
| *mclN¯* | 4.5 ± 0.4 |  | 15.5 ± 1.1 |  |
