# Supplementary figures and images for: An Autocrine Negative Feedback Loop Inhibits Dictyostelium discoideum Proliferation through Pathways Including IP3/Ca2+
Source: mBio. 2021 Jun 22;12(3):e01347-21. doi: 10.1128/mBio.01347-21 (PMC8262924; doi:10.1128/mBio.01347-21)

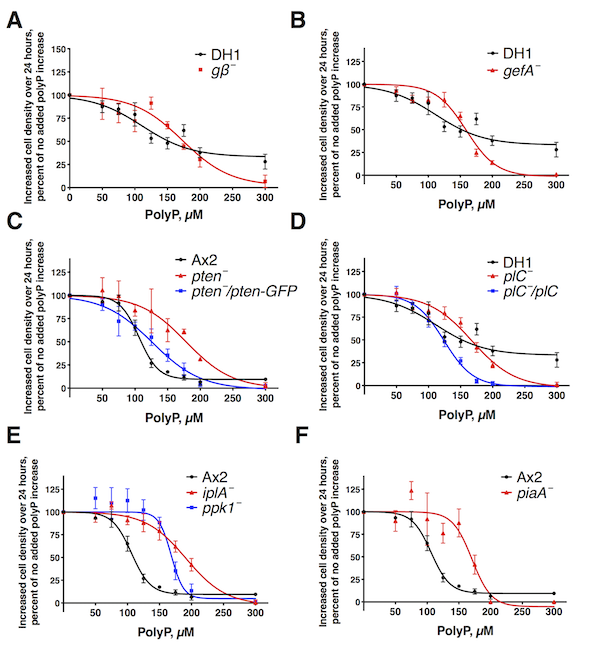

Supplement: FIG S1 [file mbio.01347-21-sf001.tif]

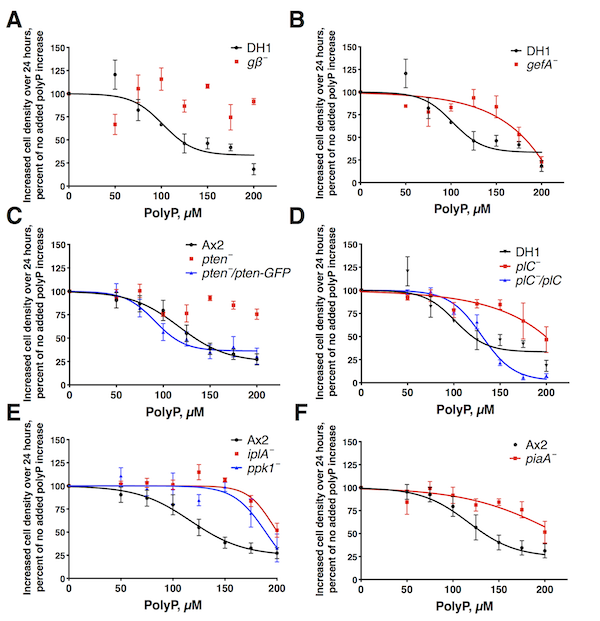

Supplement: FIG S2 [file mbio.01347-21-sf002.tif]

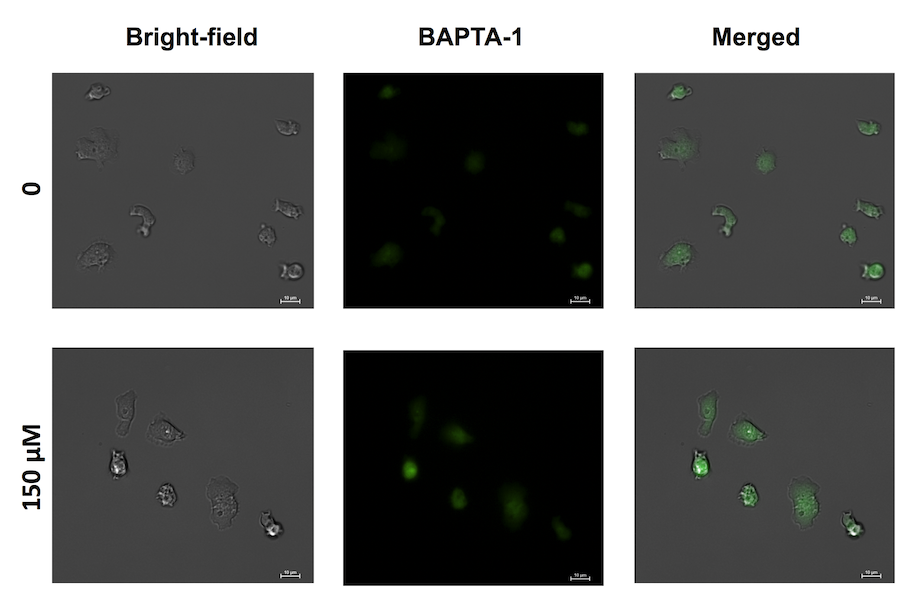

Supplement: FIG S3 [file mbio.01347-21-sf003.tif]

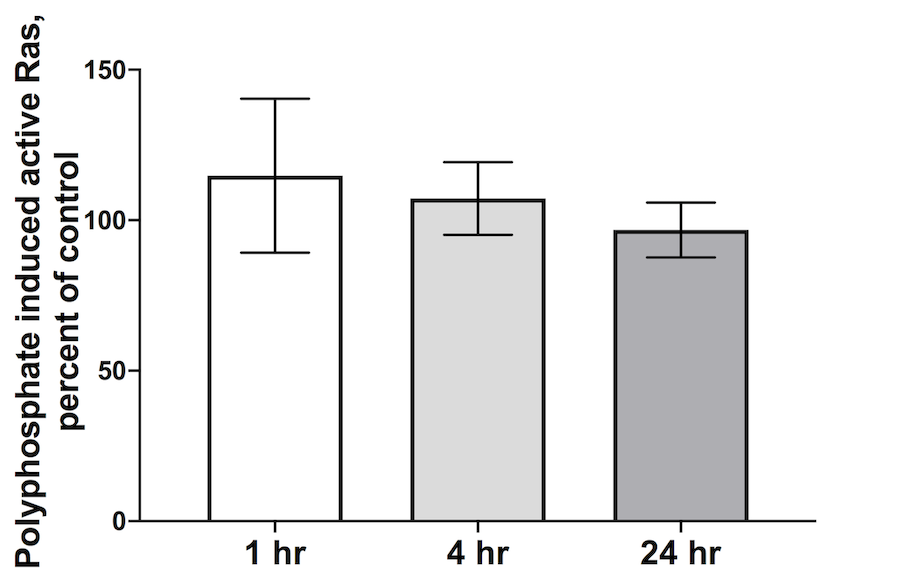

Supplement: FIG S4 [file mbio.01347-21-sf004.tif]
