# Supplementary material for: An Autocrine Negative Feedback Loop Inhibits Dictyostelium discoideum Proliferation through Pathways Including IP3/Ca2+
Source: mBio. 2021 Jun 22;12(3):e01347-21. doi: 10.1128/mBio.01347-21 (PMC8262924; doi:10.1128/mBio.01347-21)
Supplement: TABLE S1 [file mbio.01347-21-st001.docx]

**Table S1. *Dictyostelium* cell lines used in this report.**

| **Mutant Strain** | **Dictybase ID** | **Parental strain** |
| --- | --- | --- |
| *grlD*¯ | DBS0350227 | Ax2 |
| *rasC*¯ | DBS0236853 | Ax2 |
| *rasG¯* | DBS0236862 | Ax2 |
| *gefA*¯ | DBS0236896 | DH1 |
| *gβ*¯ | DBS0236531 | DH1 |
| *gα1*¯ | DBS0236088 | HPS400 |
| *gα2*¯ | DBS0236575 | DH1 |
| *gα3*¯ | DBS0235986 | HPS400 |
| *gα4*¯ | DBS0235984 | JH8 |
| *gα5*¯ | DBS0236451 | JH10 |
| *gα7*¯ | DBS0236106 | JH10 |
| *gα8*¯ | DBS0236107 | JH10 |
| *gα9*¯ | DBS0236109 | Ax3 |
| *aprA*¯ | DBS0235509 | Ax2 |
| *cfaD*¯ | DBS0302444 | Ax2 |
| *pakD*¯ | DBS0350281 | Ax2 |
| *rblA*¯ | DBS0236877 | Ax2 |
| *cnrN*¯ | DBS0302655 | Ax2 |
| *qkgA*¯ | DBS0236839 | Ax2 |
| *bzpN*¯ | DBS0349965 | Ax4 |
| *scrA*¯ | DBS0236926 | JH8 |
| *elmoE*¯ | DBS0350065 | Ax2 |
| *gcA¯/sgcA*¯ | DBS0302679 | Ax3 |
| *racC*¯ | DBS0350272 | Ax2 |
| *plA2*¯ | DBS0238068 | Ax3 |
| *pikA¯/pikB*¯ | DBS0236766 | KAx3 |
| *dagA*¯ | DBS0235559 | Ax3 |
| *pten*¯ | DBS0236830 | Ax2 |
| *pten¯/pten-GFP* | DBS0236831 | Ax2 |
| *plC*¯ | DBS0236793 | DH1 |
| *plC¯/plC* | DBS0236795 | DH1 |
| *iplA*¯ | DBS0236260 | Ax2 |
| *Dd5p4*¯ | DBS0266692 | Ax3 |
| *erk1*¯ | DBS0350622 | KAx3 |
| *erk1¯/erk2*¯ | DBS0351256 | JH10 |
| *mekA¯* | DBS0236541 | JH10 |
| *smkA*¯ | DBS0236938 | KAx3 |
| *i6kA*¯ | DBS0236426 | Ax2 |
| *ppk1*¯ | DBS0350686 | Ax2 |
| *csaA*¯ | DBS0236957 | Ax2 |
| *smlA*¯ | DBS0236939 | DH1 |
| *piaA*¯ | DBS0349879 | Ax2 |
| *lst8*¯ | DBS0236517 | KAx3 |
| *pkaC*¯ | DBS0236783 | JH10 |
| *pkcA*¯ | DBS0350916 | Ax2 |
| *amtA*¯ | DBS0235497 | Ax4 |
| *sibA¯* | DBS0236935 | DH1 |
| *tpC2¯* | DBS0350368 | DH1 |
| *trpp¯* | DBS0350368 | DH1 |
| *mcln¯* | DBS0350059 | DH1 |
| *wasA*¯ | DBS0351506 | Ax3 |
| *gdt1¯/gdt2*¯ | N/A | Ax4 |
| *gdt2*¯ | N/A | Ax4 |
| *gdt4*¯ | N/A | Ax4 |
